# Indicadores Socioeconômicos e Mortalidade por Doença Isquêmica do Coração e Doença Cerebrovascular no Brasil de 2000 a 2019

**DOI:** 10.36660/abc.20220832

**Published:** 2023-08-28

**Authors:** José Lucas Bichara, Luiz Antônio Bastos, Paolo Blanco Villela, Gláucia Maria Moraes de Oliveira

**Affiliations:** 1 Universidade Federal do Rio de Janeiro Rio de Janeiro RJ Brasil Universidade Federal do Rio de Janeiro, Rio de Janeiro, RJ – Brasil

**Keywords:** Isquemia Miocárdica, Transtornos Cerebrovasculares, Doenças Cardiovasculares, Epidemiologia

## Abstract

**Fundamento:**

Estudos prévios identificaram desigualdade na variação das taxas de mortalidade por doença isquêmica do coração (DIC) e doença cerebrovascular (DCBV) quando comparadas regiões com diferentes níveis de indicadores de desenvolvimento socioeconômico.

**Objetivo:**

Analisar a variação das taxas de mortalidade por DIC e DCBV e do desenvolvimento econômico, avaliado pelos índices sociodemográfico (ISD) e de vulnerabilidade social (IVS) no Brasil, em um período de 20 anos.

**Métodos:**

Estudo ecológico de séries temporais das taxas de mortalidade bruta e padronizada (método direto com a população brasileira de 2000) por DIC e DCBV por sexo e UF entre 2000 e 2019 comparadas com o ISD e com o IVS.

**Resultados:**

Houve melhora do ISD e IVS concomitante a redução da taxa de mortalidade padronizada por faixa etária por DIC e por DCBV no país, entretanto isso ocorreu de modo desigual entre as unidades federativas (UFs). As UFs com melhores indicadores socioeconômicos obtiveram maior redução nas taxas de mortalidade.

**Discussão:**

A variação das taxas de mortalidade por DIC e DCBV em comparação com a variação do desenvolvimento socioeconômico são compatíveis com estudos prévios, mas vamos além ao comparar de modo concomitante com o ISD e o IVS. As limitações são o fato de ser um estudo observacional, trabalhar com bancos de dados e estar sujeito ao viés ecológico.

**Conclusão:**

Os dados observados levantam a hipótese de que a melhora das condições socioeconômicas é um dos fatores responsáveis pela redução das taxas de mortalidade por DIC e DCBV.

## Introdução

O Brasil é o quinto país do mundo em extensão territorial,^1^ o sétimo país mais populoso,^2^ e de acordo com o relatório de 2019 do Programa das Nações Unidas para o Desenvolvimento (PNUD) está entre os 7 países mais desiguais do mundo.^3^ Dessa forma, torna-se terreno fértil para a análise das relações entre indicadores socioeconômicos e indicadores de saúde.

As condições socioeconômicas podem ser quantificadas por indicadores como o índice sociodemográfico (ISD) e o índice de vulnerabilidade social (IVS). O ISD é um indicador de desenvolvimento socioeconômico que possui melhor correlação com desfechos em saúde.^4^ O IVS avalia as falhas na oferta de bens e serviços públicos e atua de modo complementar aos indicadores de desenvolvimento socioeconômico.^5^ Recentemente, diversos estudos voltaram-se para essa temática, tentando compreender a relação de indicadores socioeconômicos com a mortalidade por doenças cardiovasculares (DCV).^6-15^

As DCV são as principais causas de morte no mundo e no Brasil,^6,16^ sendo as doenças isquêmicas do coração (DIC) e as doenças cerebrovasculares (DCBV), os principais responsáveis por essas estatísticas, principalmente em países de média e baixa renda.^6,16^ Estima-se que em 2019, no Brasil, as doenças cardiovasculares foram responsáveis por 27% das mortes, sendo a DIC responsável por 32,3% enquanto a DCBV representava 27,8%.^6^

As DIC e as DCBV foram extensamente estudadas, principalmente no século XX, e identificou-se que compartilham diversos fatores de risco,^17,18^ cuja análise não é suficiente para explicar as tendências de mortalidade dessas condições, quando se avaliam populações com diferentes níveis socioeconômicos. No período de 1980 a 2010 a mortalidade por DIC em regiões com alta renda como América do Norte, apresentou maior redução do que nas regiões de baixa renda, como a América do Sul.^19^ Ao avaliar a tendência de mortalidade por DCBV no período de 1996 a 2015, no Brasil, identificou-se que os estados com maior vulnerabilidade social e menor desenvolvimento humano apresentavam maior mortalidade.^15,20,21^

Este estudo propõe avaliar a relação da tendência das taxas de mortalidade por DIC e DCBV no Brasil e em suas Unidades Federativas (UFs) no período de 2000 a 2019, e a associação com a evolução do ISD e do IVS.

## Métodos

Trata-se de estudo ecológico e descritivo de séries históricas de registros de óbitos de DIC e DCBV no Brasil e em suas UFs, entre os anos de 2000 a 2019, em ambos os sexos e em todas as faixas etárias.

Os dados sobre as causas básicas de óbitos foram obtidos no site do Sistema de Informações sobre mortalidade (SIM) do Departamento de informática do Sistema único de Saúde (DATASUS) do Ministério da Saúde (MS).^22^ Foram selecionadas as informações sobre mortalidade total referentes ao Brasil e em suas UFs. Utilizaram-se como variáveis a faixa etária, o sexo e óbitos por residência. Para a pesquisa em faixas etárias, a população foi fragmentada em faixas etárias da seguinte forma: 0-19 anos, 20-29 anos e subsequentemente em faixas com 10 anos até o grupo de maiores de 80 anos.

Para seleção de óbitos cuja causa básica tenha sido DIC foi utilizado o grupo de mesmo nome no Código internacional de doenças (CID-10), o qual é representado pelos códigos I20-I25, assim como para DCBV, cujos códigos são do I60 ao I69.^23^

Sequencialmente, procedeu-se ao *download* de arquivos em formato .CSV que foram convertidos para XLS no programa Microsoft Excel, utilizado para análise de dados e construção de gráficos e tabelas.

As informações sobre a população residente no Brasil e em suas UFs foram também retiradas do *site* do DATASUS,^22^ que utiliza os dados censitários do Instituto Brasileiro de Geografia e Estatística (IBGE) de 1980, 1991, 2000 e 2010, projeções intercensitárias até 2012, e projeções populacionais de 2013 em diante.

As informações sobre o ISD foram retiradas do site do Global Health Data Exchange o qual possui o ISD calculado para o Brasil e para suas unidades federativas no período de 1950 à 2020, e as informações sobre o IVS foram obtidas no Atlas da Vulnerabilidade Social,^5^ que possui dados do Brasil e de suas UFs do período de 2000 a 2017. O IVS começou a ser calculado para a população brasileira no ano de 2000, e o ano de 2019 é o ano mais recente com dados disponíveis. Importante reforçar que ambos os indicadores variam de zero a 1, entretanto para o ISD o 1 é a situação de maior desenvolvimento e para o IVS o zero é a situação de menor vulnerabilidade.

A partir desses dados foram realizados os cálculos da taxa de mortalidade bruta e da padronizada pelo método direto, tendo como população padrão a população brasileira do ano 2000 para a DIC e para a DCBV. Avaliou-se a tendência temporal das taxas de mortalidade no período de 2000 a 2019, e a associação com o ISD e o IVS no mesmo período.

Foram construídas tabelas e calculadas as medianas e os quartis dos valores obtidos nos anos de 2000, 2009 e de 2019 para o ISD e para as taxas de mortalidade por DIC e por DCBV. Para o IVS foram escolhidos os anos de 2000, 2010 e 2017, devido a ausência de dados para os anos de 2009 e 2019.

## Resultados

No período de 2000 a 2019, ocorreram 1.925.765 mortes por DCBV e 1.968.160 mortes por DIC, no Brasil, sendo 50,54% e 58,19% respectivamente, no sexo masculino.

Na tabela 1 observa-se que o IVS variou de 0,446 no ano de 2000 à 0,243 no ano de 2017, com resultado mínimo de 0,238, no ano de 2016, enquanto o ISD variou de 0,538 em 2000 a 0,64 em 2019, apresentando aumento no período.

**Tabela 1:** Evolução do índice de vulnerabilidade social e do índice sóciodemográfico no Brasil, no período de 2000-2019

Ano	IVS	SDI
2000	0,446	0,538
2001	-	0,543
2002	-	0,547
2003	-	0,551
2004	-	0,556
2005	-	0,561
2006	-	0,566
2007	-	0,572
2008	-	0,577
2009	-	0,583
2010	0,326	0,59
2011	0,266	0,597
2012	0,249	0,603
2013	0,245	0,61
2014	0,243	0,616
2015	0,248	0,622
2016	0,238	0,627
2017	0,243	0,632
2018	-	0,636
2019	-	0,64

**Figura 1:**
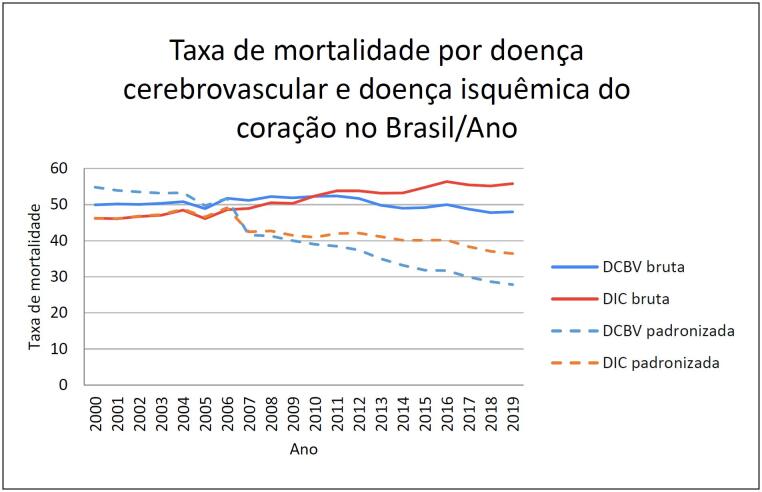
Taxas de mortalidade bruta e padronizada por doença cerebrovascular e doença isquêmica do coração no Brasil no período de 2000-2019.

Na figura 1, nota-se que no período estudado a taxa de mortalidade bruta por DCBV no Brasil apresentou pouca variação (49,89/100 mil habitantes em 2000 para 47,97/100 mil habitantes em 2019), enquanto a taxa de mortalidade por DIC variou de 46,20/100 mil habitantes para 55,80/100 mil habitantes, tornando essa a principal causa de mortalidade por DCV no país.

Na figura 1, observa-se ainda as reduções das taxas de mortalidade padronizadas, sendo de 49,81/100 mil habitantes em 2000 para 30,98/100 mil habitantes em 2019, e de 46,12/100 mil habitantes para 36,42/100 mil habitantes, para DCBV e DIC, respectivamente, no mesmo período.

Nesse período, para o sexo masculino, a taxa de mortalidade padronizada para DCBV variou de 51,62/ 100 mil habitantes para 33/100 mil habitantes, e para DIC variou de 54,33/100 mil habitantes para 44,64 por 100 mil habitantes. No sexo feminino, para DCBV, as variações foram de 48,04/100 mil habitantes em 2000 para 29,18/100 mil habitantes em 2019, e para DIC os valores foram de 38,15/100 mil habitantes e de 28,60/100 mil habitantes, respectivamente.

A tabela 2 retrata a variação do ISD no Brasil e em suas UFs no período de 2000 à 2019. Observa-se que, nesse período, o ISD variou positivamente em 17,47%, e aumentou em todas as unidades federativas com maior destaque para Tocantins, Maranhão e Piauí com os maiores aumentos proporcionais. No ano de 2019, os Estados com os melhores indicadores continuaram concentrados nas regiões Sul, Sudeste e Centro Oeste.

**Tabela 2:** Índice sociodemográfico do Brasil e de suas unidades federativas nos anos de 2000, 2010 e 2019, dividida por quartis e variação percentual no período

Índice Sociodemográfico				
UF/Ano	2000	2010	2019	Variação
**Brasil**	0,538	0,59	0,64	18,95%
Rondônia	0,48	0,547	0,606	26,25%
Acre	0,435	0,501	0,562	29,19%
Amazonas	0,505	0,548	0,602	19,20%
Roraima	0,482	0,55	0,61	26,55%
Pará	0,465	0,51	0,569	22,36%
Amapá	0,553	0,594	0,641	15,91%
Tocantins	0,422	0,514	0,583	38,15%
Maranhão	0,327	0,376	0,444	35,77%
Piauí	0,388	0,448	0,509	31,18%
Ceará	0,442	0,501	0,558	26,24%
Rio Grande do Norte	0,454	0,519	0,576	26,87%
Paraíba	0,431	0,49	0,548	27,14%
Pernambuco	0,453	0,51	0,571	26,04%
Alagoas	0,404	0,461	0,518	28,21%
Sergipe	0,473	0,532	0,583	23,25%
Bahia	0,448	0,505	0,562	17,08%
Minas Gerais	0,538	0,596	0,643	19,51%
Espírito Santo	0,543	0,607	0,66	21,54%
Rio de Janeiro	0,613	0,658	0,702	14,51%
São Paulo	0,61	0,658	0,702	15,08%
Paraná	0,555	0,615	0,662	19,27%
Santa Catarina	0,593	0,642	0,684	15,34%
Rio Grande do Sul	0,589	0,646	0,691	17,31%
Mato Grosso do Sul	0,526	0,585	0,639	21,48%
Mato Grosso	0,516	0,587	0,642	24,41%
Goiás	0,508	0,573	0,628	23,62%
Distrito Federal	0,676	0,732	0,777	14,94%
**Quartis**				
1° Quartil : 0-24%				
2° Quartil: 25-49%				
3° Quartil: 50-74%				
4° Quartil: >75%				

Na tabela 3 observa-se que o IVS no Brasil apresentou redução de 45,51% no período de 2000 a 2017 e que os Estados do Rio de Janeiro, Santa Catarina e Distrito Federal tiveram piora do indicador. Apesar disso, Santa Catarina continuou com o melhor IVS do país. Destaca-se também que os Estados de Rondônia e Tocantins foram os responsáveis pelas maiores quedas proporcionais no período. Também é importante notar que em comparação com o ano de 2000, no ano de 2017 o gradiente entre os IVS nas UFs reduziu consideravelmente, sendo de 0,57 em 2000 e de 0,24 em 2017. Apesar disso, assim como ocorreu com o ISD, ao final do período de estudo os melhores indicadores continuaram predominando nos Estados das regiões Sul, Sudeste e Centro Oeste.

**Tabela 3:** Índice de Vulnerabilidade Social no Brasil e em suas unidades federativas nos anos de 2000, 2010 e 2017 e sua variação percentual no período

Índice de Vulnerabilidade Social				
UF/Ano	2000	2010	2017	Variação
Brasil	0,446	0,326	0,243	-45,51%
Rondônia	0,493	0,319	0,191	-61,25%
Acre	0,606	0,443	0,374	-38,28%
Amazonas	0,658	0,488	0,327	-50,30%
Roraima	0,461	0,366	0,232	-49,67%
Pará	0,618	0,469	0,278	-55,01%
Amapá	0,54	0,404	0,253	-53,14%
Tocantins	0,551	0,366	0,24	-56,44%
Maranhão	0,684	0,521	0,349	-48,97%
Piauí	0,551	0,403	0,279	-49,36%
Ceará	0,53	0,378	0,272	-48,67%
Rio Grande do Norte	0,509	0,349	0,283	-44,40%
Paraíba	0,526	0,385	0,292	-44,48%
Pernambuco	0,564	0,414	0,336	-40,42%
Alagoas	0,608	0,461	0,338	-44,40%
Sergipe	0,531	0,393	0,298	-43,87%
Bahia	0,552	0,403	0,298	-46,01%
Minas Gerais	0,403	0,282	0,207	-48,63%
Espírito Santo	0,395	0,274	0,227	-42,53%
Rio de Janeiro	0,133	0,323	0,284	113%
São Paulo	0,244	0,297	0,241	-1,20%
Paraná	0,365	0,252	0,186	-49,04%
Santa Catarina	0,114	0,192	0,134	17,54%
Rio Grande do Sul	0,327	0,234	0,209	-36,08%
Mato Grosso do Sul	0,42	0,289	0,194	-53,80%
Mato Grosso	0,427	0,277	0,227	-46,83%
Goiás	0,457	0,331	0,247	-45,95%
Distrito Federal	0,173	0,294	0,258	49,13%
**Quartis**				
1° Quartil : 0-24%				
2° Quartil: 25-49%				
3° Quartil: 50-74%				
4° Quartil: >75%				

Na tabela 4, nota-se que no ano de 2000 as UFs das regiões Sul, Sudeste e Centro-Oeste eram os responsáveis pelas maiores taxas de mortalidade padronizadas por DCBV. As UFs dessas regiões apresentaram as maiores quedas percentuais no período e em 2019 possuíam taxas de mortalidade no quartil inferior do conjunto analisado. Nas regiões Norte e Nordeste apenas Rondônia, Rio Grande do Norte e Bahia alcançaram taxas de mortalidade no quartil inferior do país. É importante ressaltar que os Estados do Acre, Paraíba, Rio Grande do Norte, Piauí e Maranhão apresentaram piora considerável desse indicador, no período.

**Tabela 4:** Taxa de mortalidade por doença cerebrovascular padronizada por faixa etária e por sexo no Brasil e em suas unidades federativas nos anos de 2000, 2009 e 2019 dividida em quartis e sua variação percentual no período

Taxa de mortalidade por doença cerebrovascular padronizada por faixa etária				
UF/Ano	2000	2009	2019	Variação
Brasil	49,81	42,27	30,98	-37,80%
Rondônia	50,01	42,42	30,26	-39,49%
Acre	34,33	42,92	37,83	10,19%
Amazonas	37,7	35,84	37,48	-0,01%
Roraima	58,69	37,02	57,7	-0,01%
Pará	38,06	45,21	40,4	0,06%
Amapá	49,26	36,84	39,07	-20,68%
Tocantins	39,17	50,79	38,04	-0,03%
Maranhão	26,26	52,82	44,61	69,87%
Piauí	39,17	62,69	50,45	28,79%
Ceará	38,13	45,24	34,16	-10,41%
Rio Grande do Norte	25,2	36,12	28,45	12,89%
Paraíba	28,63	43,75	32,3	12,81%
Pernambuco	48,92	45,78	37,92	-22,48%
Alagoas	48,79	57,6	47,72	-2,19%
Sergipe	39,65	46,78	36,7	-7,44%
Bahia	34,03	36,78	31,73	-6,75%
Minas Gerais	48,1	36,14	26,46	-44,98%
Espírito Santo	60,86	48,85	31,62	-48,04%
Rio de Janeiro	62,21	45,29	30,32	-51,26%
São Paulo	54,68	39,52	27,57	-49,57%
Paraná	65,78	47,17	31,87	-51,55%
Santa Catarina	56,74	38,46	26,51	-53,27%
Rio Grande do Sul	60,11	46,53	29,98	-50,12%
Mato Grosso do Sul	57,26	43,9	32,92	-42,50%
Mato Grosso	56,85	42,8	31,17	-45,17%
Goiás	48,65	37,15	31,43	-35,39%
Distrito Federal	56,77	37,5	25,08	-55,82%
Quartis				
1° Quartil : 0-24%				
2° Quartil: 25-49%				
3° Quartil: 50-74%				
4° Quartil: >75%				

Na tabela 5, observa-se a evolução da taxa de mortalidade por DIC padronizada no Brasil e em suas UFs no período estudado. Nota-se que em 2000 a grande maioria dos Estados das regiões Norte e Nordeste possuíam taxas de mortalidade muito abaixo da média nacional, enquanto as UFs das regiões Sul, Sudeste e Centro-Oeste concentravam as maiores taxas de mortalidade por DIC do país. No período em estudo, todas as UFs das regiões Sul, Sudeste e Centro-Oeste apresentaram redução dessa taxa, enquanto nas regiões Norte e Nordeste isso não foi observado. Ao fim do período, as maiores taxas de mortalidade por DIC estavam concentradas nos Estados das regiões Norte e Nordeste do país. Importante destacar que os Estados de Roraima, Acre, Paraíba e Maranhão mais que dobraram suas taxas de mortalidade.

**Tabela 5:** Taxa de mortalidade por doença isquêmica do coração padronizada por faixa etária e por sexo no Brasil e em suas unidades federativas nos anos de 2000, 2009 e 2019 divididas por quartis e suas variações percentuais no período

	Taxa de mortalidade por doença isquêmica do coração padronizada por faixa etária			
UF/Ano	2000	2009	2019	Variação
Brasil	46,12	41,46	36,42	-21,03%
Rondônia	32,49	38,53	30,97	-4,67%
Acre	21,31	29,43	45,42	113%
Amazonas	19,51	26,44	28,54	46,28%
Roraima	20,12	26,85	40,41	100,80%
Pará	23,8	31,15	36,6	53,78%
Amapá	17,51	21,98	35,48	102%
Tocantins	27,73	42,19	38,61	39,23%
Maranhão	12,95	36,97	44,33	242%
Piauí	23,9	41,12	45,45	90,16%
Ceará	23,12	35,35	41,32	78,71%
Rio Grande do Norte	32,02	44,02	49,25	53,81%
Paraíba	15,37	43,81	45,44	195%
Pernambuco	47,33	60,16	50,75	7,22%
Alagoas	25,26	37,72	46,08	82,42%
Sergipe	17,25	30,93	30,85	78,84%
Bahia	21,72	26,16	26,69	21,04%
Minas Gerais	36,14	29,51	23,26	-35,63%
Espírito Santo	43,66	51,61	37,03	-15,18%
Rio de Janeiro	57,22	46,39	41,88	-26,80%
São Paulo	65,03	47,94	40,94	-37,04%
Paraná	60,87	43,9	31,45	-48,33%
Santa Catarina	51,17	41,01	30,93	-39,55%
Rio Grande do Sul	71,05	46,3	31,01	-56,35%
Mato Grosso do Sul	54,47	50,79	49,77	-8,62%
Mato Grosso	38,05	37,67	32,38	-14,90%
Goiás	39,93	39,3	37,24	-6,73%
Distrito Federal	49,32	30,15	23,91	-51,52%
**Quartis**				
1° Quartil : 0-24%				
2° Quartil: 25-49%				
3° Quartil: 50-74%				
4° Quartil: >75%				

**Figura 2:**
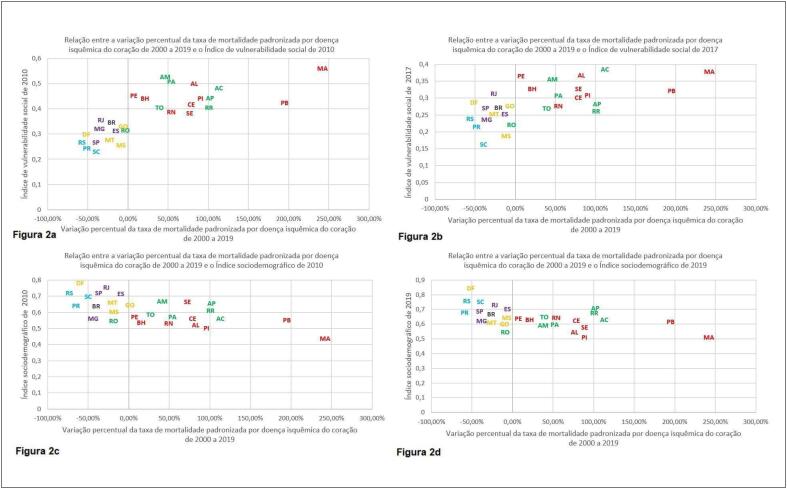
Comparações da variação percentual da taxa de mortalidade padronizada por doença isquêmica do coração de 2000 a 2019 com o Índice de vulnerabilidade social de 2010 (a), com o Índice de vulnerabilidade social de 2017 (b), com o Índice sociodemográfico de 2010 (c) e com o Índice sociodemográgico de 2019(d).

**Figura 3:**
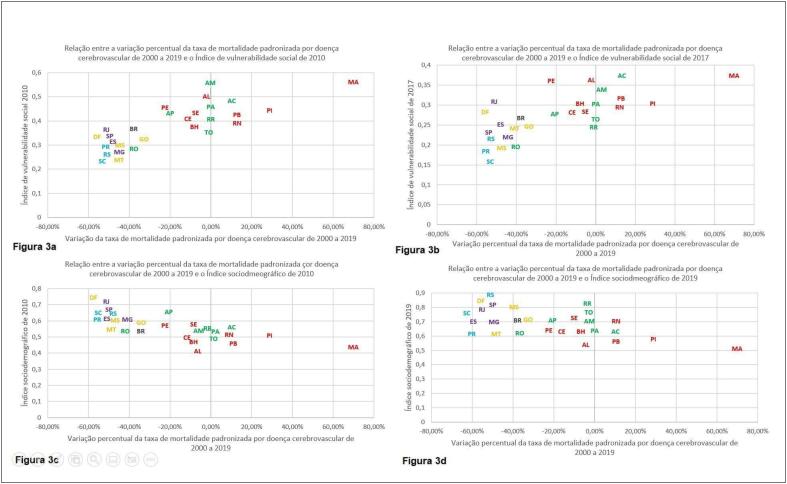
Comparações da variação percentual da taxa de mortalidade padronizada por doença cerebrovascular de 2000 a 2019 com o índice de vulnerabilidade social de 2010 (a), com o Índice de vulnerabilidade social de 2017 (b), com o Índice sociodemográfico de 2010 (c) e com o Índice sociodemográfico de 2019 (d).

Foi realizada ainda a comparação entre a variação percentual das taxas de mortalidade padronizadas e o ISD de 2010 e de 2019, o IVS de 2010 e de 2017 e a variação percentual do ISD e do IVS durante todo o período. Na figura 2a observa-se a comparação entre a variação percentual da taxa de mortalidade padronizada por DIC de 2000 a 2019 e o IVS de 2010, já na figura 3a é observada a comparação da variação de taxa de mortalidade padronizada por DCBV de 2000 a 2019 e o IVS de 2010. Nas figuras 2b e 3b, comparam-se, respectivamente, as mesmas variações percentuais com o IVS de 2017. Nas figuras 2c e 3c, foram realizadas as comparações das variações das taxas de mortalidade padronizadas por DIC e DCBV, respectivamente, com o ISD de 2010 e nas figuras 2d e 3d, com o ISD de 2019.

**Figura 4:**
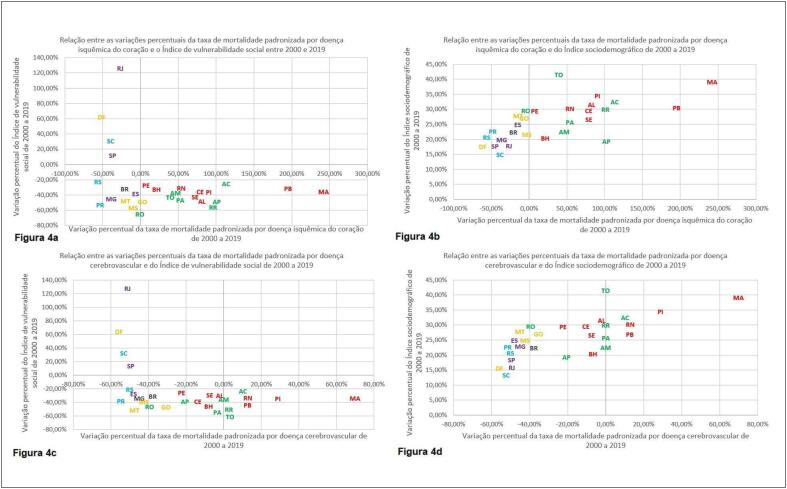
Comparações da variação percentual da taxa de mortalidade padronizada por doença isquêmica do coração no período de 2000 a 2019 com a variação percentual do Índice de vulnerabilidade social (a) e do Índice sociodemográfico (b) no mesmo período; Comparações da variação percentual da taxa de mortalidade padronizada por doença cerebrovascular no período de 2000 a 2019 com a variação percentual do Índice de vulnerabilidade social (c) e do Índice sociodemográfico (d) no mesmo período.

Foram realizadas também comparações entre as variações percentuais das taxas de mortalidades padronizadas pela faixa etária por DIC e DCBV e as variações percentuais do IVS e do ISD no período de 2000 a 2019, conforme exposto na Figura 4.

## Discussão

No presente estudo, foi possível observar que, no período estudado, a DIC se tornou a principal causa de mortalidade no país, concordante com o demonstrado em estudos prévios.^6,8,24^ Além disso, embora as taxas de mortalidade brutas por DIC e DCBV tenham aumentado, devido a transição demográfica que ocorre no país, houve redução da taxa de mortalidade padronizada pela faixa etária para ambas as condições. Observou-se também a melhora do ISD e do IVS, com predomínio dos melhores indicadores nas UFs das regiões Sul, Sudeste e Centro-Oeste. Ao comparar as variações percentuais das taxas de mortalidade padronizadas por DIC e DCBV com o IVS de 2010 e de 2017 e o ISD de 2010 e de 2019, observou-se que as UFs com melhores indicadores apresentaram maior redução percentual da mortalidade. Por fim, quando comparadas as variações percentuais do ISD no período com as variações percentuais das taxas de mortalidade padronizadas por DIC e DCBV notou-se que as UFs das regiões Sul, Sudeste e Centro-Oeste tiveram maior redução percentual das taxas de mortalidade, apesar de menor variação percentual do ISD, o que pode ter ocorridos pois as UFs dessas regiões já partiram de valores de ISD relativamente elevados. Entretanto, não foi possível identificar um padrão quando essa comparação foi realizada com a variação percentual do IVS, sendo necessário destacar que as UFs de Rio de Janeiro, São Paulo, Distrito Federal e Santa Catarina apresentaram piora do IVS.

Importante notar que algumas UFs das regiões Norte e Nordeste apresentaram importante variação positiva do ISD, e variação negativa do IVS, mas que não se traduziram em redução da mortalidade por DIC e DCBV, sugerindo que pode haver um valor mínimo necessário a ser alcançado para que os efeitos sejam observados. Efeito esse que já foi identificado com outro índice de desenvolvimento humano médio (IDHM).^7^

Esse estudo diferencia-se de outros estudos publicados até o momento sobre o tema^7,8,10,15,19,20^ pois se propõem a analisar a comparação das taxas de mortalidade por DIC e DCBV associada com 2 indicadores socioeconômicos que atuam de modo complementar, visto que o SDI avalia o grau de desenvolvimento social de um país ou região, enquanto o IVS cumpre o papal de identificar as regiões mais vulneráveis. Além disso, foi feita uma análise da variação percentual das taxas e dos indicadores com o intuito de comparar cada UF a si própria, algo até então não demonstrado.

A compreensão fisiopatológica das influências dos fatores de risco clássicos nas doenças cardiovasculares como hipertensão arterial sistêmica, dislipidemia, diabetes mellitus, obesidade, tabagismo e sedentarismo foram e são fundamentais,^17,18^ para guiar a as medidas de prevenção e redução da mortalidade. Entretanto, estudos prévios em âmbito global, identificaram diferenças nas tendências de taxas de mortalidade por DIC e DCBV entre os países com diferentes níveis socioeconômicos.^10,23,24^ Inclusive, alguns estudos demonstraram que países de maior nível socioeconômico apresentam maior incidência de doenças não comunicáveis, grupo que inclui as DCVs, devido a maior exposição a fatores de risco clássicos e maior disponibilidade de métodos diagnóstico e terapêuticos. Apesar disso, a probabilidade de ocorrência de óbitos por essas condições é maior em países de menor nível socioeconômico.^11,12,25,26^

No âmbito da DCBV, estudos prévios também já haviam identificado a tendência de redução de mortalidade no país.^7,8,13,27,28^ Além disso, já havia sido identificado, também, que essa redução estava ocorrendo de modo heterogêneo, sendo que as regiões Norte e Nordeste apresentaram reduções menos evidentes e algumas UFs que, inclusive apresentaram aumento dessa mortalidade.^7,8,29^ Com relação a DIC, padrão semelhante também já havia sido observado previamente.^9,21,24^

Condições que contribuíram para melhora das taxas de mortalidade por essas condições em âmbito nacional incluem o maior acesso a serviços de saúde e adoção de estratégias de prevenção,^7,8,30,31^ com a expansão da atenção primária a saúde^7,32,33^ e o desenvolvimento do plano de ações estratégicas para o enfrentamento das doenças crônicas não transmissíveis.^7,34^ No caso específico da DCBV, houve ainda a organização da rede de atendimento inicial ao AVC.^7,35,36^ Entretanto, essas melhoras ocorreram de modo heterogêneo no território nacional. Soma-se a isso, o fato dos grandes centros, que se localizam principalmente nas regiões Sul e Sudeste, terem acesso a maior disponibilidade de tratamentos medicamentosos e intervencionistas,^6^ melhores índices de escolaridade e de desenvolvimento humano.^8,9,14,37^

Entre as justificativas plausíveis para a piora das taxas mortalidade por DIC e DCBV em UFs das regiões Norte e Nordeste, apesar da melhora dos indicadores socioeconômicos, podemos destacar: Subnotificação da mortalidade por essas condições que é maior nessas regiões, principalmente, no início do período analisado^7,38^ e a transição demográfica ocorrendo de modo mais tardio nas regiões Norte e Nordeste do país.^7,39^

As principais limitações do estudo incluem o fato de ser um estudo observacional, portanto atua como um gerador de hipóteses. Além disso, baseia-se em banco de dados, dessa forma, está sujeito a vieses por falhas na coleta de dados: subnotificação, causas mal definidas ou *garbage codes.* Entretanto, são limitações que atuam de forma sistêmica, em todas as declarações e bancos de óbito, de forma a não transformar a limitação em impedimento para a análise global dos dados.

Por isso, os resultados apresentados neste trabalho somam-se com os estudos prévios^7-9,19,20^ sobre o tema ao sugerir que a melhora das condições socioeconômicas, como renda, trabalho, educação e acesso à infraestrutura urbana, têm impacto na redução da mortalidade por DCV.

## Conclusão

A análise concomitante do ISD e do IVS permitiu uma avaliação mais abrangente do perfil socioeconômico do Brasil e de suas UFs e da avaliação de uma possível relação desses indicadores com a mortalidade por DIC e DCBV. Observou-se que o país apresentou melhora do desenvolvimento humano e redução da vulnerabilidade social, associado a redução das taxas de mortalidade padronizadas por faixa etária por DIC e DCBV. Entretanto, isso ocorreu de modo heterogêneo em seu território. Os melhores indicadores socioeconômicos e as menores taxas de mortalidade por DIC e DCBV ficaram concentradas nas regiões Sul, Sudeste e Centro-Oeste do país. Tal resultado, sugere que maior desenvolvimento social e menor vulnerabilidade social podem estar relacionados com menor mortalidade por DIC e DCBV, embora não tenha sido possível identificar uma relação direta entre os indicadores e as taxas de mortalidade no presente estudo.
