# Supplementary material for: Indicadores Socioeconômicos e Mortalidade por Doença Isquêmica do Coração e Doença Cerebrovascular no Brasil de 2000 a 2019
Source: Arq Bras Cardiol. 2023 Aug 28;120(8):e20220832. [Article in Portuguese] doi: 10.36660/abc.20220832 (PMC10519229; doi:10.36660/abc.20220832)
Supplement: Translation

# Translation: Socioeconomic Indicators and Mortality from Ischemic Heart Disease and Cerebrovascular Disease in Brazil from 2000 to 2019

**Authors:** José Lucas Bichara, Luiz Antônio Bastos, Paolo Blanco Villela, Gláucia Maria Moraes de Oliveira

**Affiliations:** 1 Rio de Janeiro RJ Brazil Universidade Federal do Rio de Janeiro, Rio de Janeiro, RJ - Brazil

**Keywords:** Myocardial ischemia, Cerebrovascular Disorders, Cardiovascular Disease, Epidemiology

## Abstract

**Background:**

Previous studies have identified inequalities in the variation of mortality rates from ischemic heart disease (IHD) and cerebrovascular disease (CBVD) when comparing regions with different levels of socioeconomic development indicators.

**Objective:**

To analyze the variation in IHD and CBVD mortality rates and economic development, evaluated by the sociodemographic index (SDI) and social vulnerability index (SVI) in Brazil over a period of 20 years.

**Methods:**

Ecological study of time series of crude and standardized mortality rates (direct method, based on the Brazilian population in year 2000) from IHD and CBVD by sex and Federative Unit (FU) between 2000 and 2019, compared using the SDI and SVI.

**Results:**

There was an improvement in SDI and SVI concomitantly to a reduction in age-standardized mortality rate from IHD and _CBVD_ in the country; however, this occurred unevenly across the FUs. The FUs with the best socioeconomic indicators had the greatest reduction in mortality rates.

**Discussion:**

The variations in mortality rates from IHD and CBVD, compared using variations in socioeconomic development, are aligned with those from previous studies, but the present study goes further by including the indicators SDI and SVI in the comparison. The limitations include the observational nature of the study, the use of databases, and the vulnerability to ecological bias.

**Conclusion:**

The observed data raise the hypothesis that the improvement in socioeconomic conditions is one of the factors responsible for the reduction in mortality rates from _IHD_ and _CBVD_.

## Introduction

Brazil is the fifth country in territorial area^1^ and the seventh most populous country.^2^ According to the 2019 report by the United Nations Development Program (UNDP), Brazil is among the seven most unequal countries worldwide.^3^ Thus, the country is a fertile ground for analyses of the relationships between socioeconomic indicators and health indicators.

Socioeconomic conditions can be quantified by indicators like the sociodemographic index (SDI) and the social vulnerability index (SVI). The SDI is an indicator of socioeconomic development that correlates better with health outcomes.^4^ The SVI evaluates failures in the supply of public goods and services and complements socioeconomic development indicators.^5^ Recently, several studies focused on this topic in an attempt to elucidate the relationship between socioeconomic indicators and mortality from cardiovascular diseases (CVDs).^6-15^

CVDs are the main causes of death in Brazil and worldwide,^6,16^ and ischemic heart diseases (IHDs) and cerebrovascular diseases (CBVDs) are the main conditions responsible for these statistics, especially in countries with medium and low income.^6,16^ According to estimates, CVDs were responsible for 27% of the deaths in Brazil in 2019, with IHDs accounting for 32.3% and CBVDs for 27.8% of them.^6^

Both IHDs and CBVDs have been extensively studied, particularly in the 20th century, and have been identified as sharing several risk factors,^17,18^ but these analyses are not sufficient to explain the mortality trends of these conditions when populations with different socioeconomic levels are evaluated. In the period from 1980 to 2010, mortality from IHD showed a greater reduction in high-income regions like North America than in low-income regions like South America.^19^ When the CBVD mortality trend was evaluated from 1996 to 2015 in Brazil, states with greater social vulnerability and lower human development were identified as having higher mortality.^15,20,21^

This study proposes to evaluate the relationship between the trend in mortality rates from IHD and CBVD in Brazil and its Federative Units (FUs) in the period from 2000 to 2019 and the association with the evolution of SDI and SVI.

## Methods

This is an ecological and descriptive study of historical series of death records from IHD and CBVD in Brazil and its FUs between the years 2000 and 2019, in both sexes and across all age groups.

Data related to the underlying causes of death were obtained from the Mortality Information System (*Sistema de Informações sobre Mortalidade*, SIM) of the Department of Informatics of the Unified Health System (*Departamento de Informática do Sistema Único de Saúde*, DATASUS) of the Ministry of Health (*Ministério da Saúde*, MS).^22^ Information on total mortality in Brazil and its FUs was selected. Age group, sex, and deaths per residence location were used as variables. For the age group analysis, the population was stratified into age groups as follows: 0–19 years, 20–29 years, and, subsequently, in groups of 10 years up to the age above 80 years.

For the selection of deaths in which the underlying cause was IHD, the group with this same name in the International Code of Diseases (ICD-10) was used, which is represented by codes I20–I25; the same was done for CBVD, which is represented by codes I60–I69.^23^

Sequentially, the files were downloaded in a .CSV format and converted into .XLS using the software Microsoft Excel, which was used for data analysis and construction of graphs and tables.

The information on the population residing in Brazil and its FUs was also retrieved from the DATASUS^22^ website, which uses census data from the Brazilian Institute of Geography and Statistics (*Instituto Brasileiro de Geografia e Estatística*, IBGE) from 1980, 1991, 2000, and 2010, intercensus projections up to 2012, and population projections from 2013 onwards.

Information on SDI was obtained from the website of the Global Health Data Exchange, which displays SDI calculated for Brazil and its FUs in the period from 1950 to 2020, while the information on SVI was obtained from the Social Vulnerability Atlas (*Atlas da Vulnerabilidade Social*),^5^ which displays data from Brazil and its FUs from 2000 to 2017. The calculation of the SVI in the Brazilian population began in the year 2000, and the most recent year with available data is 2019. Of note, both indicators range from 0 to 1, in which for SDI, 1 indicates a situation of greatest development, while for SVI, 0 indicates a situation of least vulnerability.

Based on these data, the crude and standardized mortality rates were calculated by the direct method, using the Brazilian population in the year 2000 as the standard population for IHD and CBVD. The temporal trend in mortality rates from 2000 to 2019 was evaluated, along with their association with SDI and SVI in the same period.

Tables were constructed, and the medians and quartiles of the values obtained in the years 2000, 2009, and 2019 were calculated for the SDI and for the IHD and CBVD mortality rates. For SVI, the years 2000, 2010, and 2017 were chosen due to the lack of data for the years 2009 and 2019.

## Results

In the period from 2000 to 2019 in Brazil, there were 1,925,765 deaths from CBVD and 1,968,160 deaths from IHD, of which 50.54% and 58.19%, respectively, occurred in the male sex.

As shown in table 1, the SVI ranged from 0.446 in the year 2000 to 0.243 in the year 2017, with a minimum value of 0.238 in the year 2016, while the SDI ranged from 0.538 in 2000 to 0.64 in 2019, indicating an increase in the period.

**Table 1:** Evolution of the social vulnerability index and the sociodemographic index in Brazil in the period of 2000 to 2019

Year	IVS	SDI
2000	0.446	0.538
2001	-	0.543
2002	-	0.547
2003	-	0.551
2004	-	0.556
2005	-	0.561
2006	-	0.566
2007	-	0.572
2008	-	0.577
2009	-	0.583
2010	0.326	0.59
2011	0.266	0.597
2012	0.249	0.603
2013	0.245	0.61
2014	0.243	0.616
2015	0.248	0.622
2016	0.238	0.627
2017	.	0.632
2018	-	0.636
2019	-	0.64

Figure 1 shows that the crude mortality rate from CBVD in Brazil showed a small variation in the analyzed period (49.89/100 thousand inhabitants in 2000 to 47.97/100 thousand inhabitants in 2019), while the mortality rate from IHD ranged from 46.20/100 thousand inhabitants to 55.80/100 thousand inhabitants, making this the leading cause of mortality from CVD in the country.

**Figure 1:** Crude and standardized mortality rates from cerebrovascular disease and ischemic heart disease in Brazil in the 2000–2019 period.

Figure 1 also shows reductions in standardized mortality rates from 49.81/100 thousand inhabitants in 2000 to 30.98/100 thousand inhabitants in 2019 for CBVD and from 46.12/100 thousand inhabitants to 36.42/100 thousand inhabitants for IHD in the same period.

During this period, in the male sex, the standardized mortality rate ranged from 51.62/100 thousand inhabitants to 33/100 thousand inhabitants for CBVD and from 54.33/100 thousand inhabitants to 44.64 per 100 thousand inhabitants for IHD. In the female sex, the variations ranged from 48.04/100 thousand inhabitants in 2000 to 29.18/100 thousand inhabitants in 2019 for CBVD and from 38.15/100 thousand inhabitants to 28.60/100 thousand inhabitants for IHD.

Table 2 shows the SDI variation in Brazil and its FUs in the period from 2000 to 2019. In the period, the SDI varied positively by 17.47% and increased across all FUs, with particular emphasis for Tocantins, Maranhão, and Piauí, which had the greatest proportional increases. In 2019, the states with the best indicators remained concentrated in the South, Southeast, and Midwest regions.

**Table 2:** Sociodemographic index in Brazil and its Federative Units in the years 2000, 2010, and 2019, divided by quartiles and percentage variation in the period

Sociodemographic index				
FU/Year	2000	2010	2019	Variation
Brazil	0.538	0.59	0.64	18.95%
Rondônia	0.48	0.547	0.606	26.25%
Acre	0.435	0.501	0.562	29.19%
Amazonas	0.505	0.548	0.602	19.2%
Roraima	0.482	0.55	0.61	26.55%
Pará	0.465	0.51	0.569	22.36%
Amapá	0.553	0.594	0.641	15.91%
Tocantins	0.422	0.514	0.583	38.15%
Maranhão	0.327	0.376	0.444	35.77%
Piauí	0.388	0.448	0.509	31.18%
Ceará	0.442	0.501	0.558	26.24%
Rio Grande do Norte	0.454	0.519	0.576	26.87%
Paraíba	0.431	0.49	0.548	27.14%
Pernambuco	0.453	0.51	0.571	26.04%
Alagoas	0.404	0.461	0.518	28.21%
Sergipe	0.473	0.532	0.583	23.25%
Bahia	0.448	0.505	0.562	17.08%
Minas Gerais	0.538	0.596	0.643	19.51%
Espírito Santo	0.543	0.607	0.66	21.54%
Rio de Janeiro	0.613	0.658	0.702	14.51%
São Paulo	0.61	0.658	0.702	15.08%
Paraná	0.555	0.615	0.662	19.27%
Santa Catarina	0.593	0.642	0.684	15.34%
Rio Grande do Sul	0.589	0.646	0.691	17.31%
Mato Grosso do Sul	0.526	0.585	0.639	21.48%
Mato Grosso	0.516	0.587	0.642	24.41%
Goiás	0.508	0.573	0.628	23.62%
Distrito Federal	0.676	0.732	0.777	14.94%
**Quartiles**				
1° Quartil : 0-24%				
2° Quartil: 25-49%				
3° Quartil: 50-74%				
4° Quartil: >75%				

Table 3 shows that the SVI in Brazil reduced by 45.51% from 2000 to 2017 and that this indicator worsened in the states of Rio de Janeiro and Santa Catarina and in the Federal District. Despite this, Santa Catarina continued with the best SVI in the country. Of note, the states of Rondônia and Tocantins accounted for the largest proportional declines in the period. Also, relative to the year 2000, the difference between the SVI in the FUs reduced considerably in 2017, from 0.57 in 2000 to 0.24 in 2017. Despite this, and as with the SDI, the best indicators continued to predominate in the states in the South, Southeast, and Midwest regions at the end of the study period.

**Table 3:** Social vulnerability index in Brazil and its Federative Units in the years 2000, 2010, and 2017 and its percentage variation in the period

	Socio vulnerability index			
FU/Year	2000	2010	2017	Variation
Brazil	0.446	0.326	0.243	-45.51%
Rondônia	0.493	0.319	0.191	-61.25%
Acre	0.606	0.443	0.374	-38.28%
Amazonas	0.658	0.488	0.327	-50.3%
Roraima	0.461	0.366	0.232	-49.67%
Pará	0.618	0.469	0.278	-55.01%
Amapá	0.54	0.404	0.253	-53.14%
Tocantins	0.551	0.366	0.24	-56.44%
Maranhão	0.684	0.521	0.349	-48.97%
Piauí	0.551	0.403	0.279	-49.36%
Ceará	0.53	0.378	0.272	-48.67%
Rio Grande do Norte	0.509	0.349	0.283	-44.4%
Paraíba	0.526	0.385	0.292	-44.48%
Pernambuco	0.564	0.414	0.336	-40.42%
Alagoas	0.608	0.461	0.338	-44.4%
Sergipe	0.531	0.393	0.298	-43.87%
Bahia	0.552	0.403	0.298	-46.01%
Minas Gerais	0.403	0.282	0.207	-48.63%
Espírito Santo	0.395	0.274	0.227	-42.53%
Rio de Janeiro	0.133	0.323	0.284	113%
São Paulo	0.244	0.297	0.241	-1.2%
Paraná	0.365	0.252	0.186	-49.04%
Santa Catarina	0.114	0.192	0.134	17.54%
Rio Grande do Sul	0.327	0.234	0.209	-36.08%
Mato Grosso do Sul	0.42	0.289	0.194	-53.8%
Mato Grosso	0.427	0.277	0.227	-46.83%
Goiás	0.457	0.331	0.247	-45.95%
Distrito Federal	0.173	0.294	0.258	49.13%
**Quartiles**				
1° Quartil : 0-24%				
2° Quartil: 25-49%				
3° Quartil: 50-74%				
4° Quartil: >75%				

Table 4 shows that in the year 2000, the FUs in the South, Southeast, and Midwest regions were responsible for the highest standardized mortality rates from CBVD. The FUs in these regions showed the highest percentage decreases in the period and, in 2019, had mortality rates in the lowest quartile in the analyzed set. In the North and Northeast regions, only Rondônia, Rio Grande do Norte, and Bahia had mortality rates in the lowest quartile of the country. Notably, this indicator deteriorated considerably during the period in the States of Acre, Paraíba, Rio Grande do Norte, Piauí, and Maranhão.

**Table 4:** Mortality rate from cerebrovascular disease standardized by age group and sex in Brazil and its Federative Units in the years 2000, 2009, and 2019 divided into quartiles and its percentage variation in the period

Mortality rate from cerebrovascular disease standardized by age group				
FU/Year	2000	2009	2019	Variation
Brazil	49.81	42.27	30.98	-37.8%
Rondônia	50.01	42.42	30.26	-39.49%
Acre	34.33	42.92	37.83	10.19%
Amazonas	37.7	35.84	37.48	-0.1%
Roraima	58.69	37.02	57.7	-0.01%
Pará	38.06	45.21	40.4	0.06%
Amapá	49.26	36.84	39.07	-20.68%
Tocantins	39.17	50.79	38.04	-0.03%
Maranhão	26.26	52.82	44.61	69.87%
Piauí	39.17	62.69	50.45	28.79%
Ceará	38.13	45.24	34.16	-10.41%
Rio Grande do Norte	25.2	36.12	28.45	12.89%
Paraíba	28.63	43.75	32.3	12.81%
Pernambuco	48.92	45.78	37.92	-22.48%
Alagoas	48.79	57.6	47.72	-2.19%
Sergipe	39.65	46.78	36.7	-7.44%
Bahia	34.03	36.78	31.73	-6.75%
Minas Gerais	48.1	36.14	26.46	-44.98%
Espírito Santo	60.86	48.85	31.62	-48.04%
Rio de Janeiro	62.21	45.29	30.32	-51.26%
São Paulo	54.68	39.52	27.57	-49.57%
Paraná	65.78	47.17	31.87	-51.55%
Santa Catarina	56.74	38.46	26.51	-53.27%
Rio Grande do Sul	60.11	46.53	29.98	-50.12%
Mato Grosso do Sul	57.26	43.9	32.92	-42.5%
Mato Grosso	56.85	42.8	31.17	-45.17%
Goiás	48.65	37.15	31.43	-35.39%
Distrito Federal	56.77	37.5	25.08	-55.82%
**Quartiles**				
1° Quartil : 0-24%				
2° Quartil: 25-49%				
3° Quartil: 50-74%				
4° Quartil: >75%				

Table 5 shows the evolution of the standardized mortality rate from IHD in Brazil and its FUs in the analyzed period. Notably, in 2000, most states in the North and Northeast regions had mortality rates far below the national average, while the FUs in the South, Southeast, and Midwest regions concentrated the highest mortality rates from IHD in the country. During the study period, all FUs in the South, Southeast, and Midwest regions showed a reduction in this rate, while the same was not observed in the North and Northeast regions. At the end of the period, the highest mortality rates from IHD were concentrated in the states of the North and Northeast regions of the country. Of note, the mortality rates more than doubled in the states of Roraima, Acre, Paraíba, and Maranhão.

**Table 5:** Mortality rate from ischemic heart disease standardized by age group and sex in Brazil and its Federative Units in the years 2000, 2009, and 2019 divided by quartiles and their percentage variations in the period

	Mortality rate from ischemic heart disease standardized by age group			
FU/Year	2000	2009	2019	Variation
Brazil	46.12	41.46	36.42	-21.03%
Rondônia	32.49	38.53	30.97	-4.67%
Acre	21.31	29.43	45.42	113%
Amazonas	19.51	26.44	28.54	46.28%
Roraima	20.12	26.85	40.41	100.8%
Pará	23.8	31.15	36.6	53.78%
Amapá	17.51	21.98	35.48	102%
Tocantins	27.73	42.19	38.61	39.23%
Maranhão	12.95	36.97	44.33	242%
Piauí	23.9	41.12	45.45	90.16%
Ceará	23.12	35.35	41.32	78.71%
Rio Grande do Norte	32.02	44.02	49.25	53.81%
Paraíba	15.37	43.81	45.44	195%
Pernambuco	47.33	60.16	50.75	7.22%
Alagoas	25.26	37.72	46.08	82.42%
Sergipe	17.25	30.93	30.85	78.84%
Bahia	21.72	26.16	26.69	21.04%
Minas Gerais	36.14	29.51	23.26	-35.63%
Espírito Santo	43.66	51.61	37.03	-15.18%
Rio de Janeiro	57.22	46.39	41.88	-26.8%
São Paulo	65.03	47.94	40.94	-37.04%
Paraná	60.87	43.9	31.45	-48.33%
Santa Catarina	51.17	41.01	30.93	-39.55%
Rio Grande do Sul	71.05	46.3	31.01	-56.35%
Mato Grosso do Sul	54.47	50.79	49.77	-8.62%
Mato Grosso	38.05	37.67	32.38	-14.9%
Goiás	39.93	39.3	37.24	-6.73%
Distrito Federal	49.32	30.15	23.91	-51.52%
**Quartiles**				
1° Quartil : 0-24%				
2° Quartil: 25-49%				
3° Quartil: 50-74%				
4° Quartil: >75%				

Percentage variations in standardized mortality rates were also compared with the SDI in 2010 and 2019, the SVI in 2010 and 2017, and with percentage variations in SDI and SVI over the entire period. Figure 2a shows a comparison between the percentage variation in the standardized mortality rate from IHD from 2000 to 2019 and the SVI in 2010, while figure 3a shows a comparison of the variation in the standardized mortality rate from CBVD from 2000 to 2019 and the SVI in 2010. Figures 2b and 3b, respectively, compare the same percentage variations with the SVI in 2017. Comparisons of variations in standardized mortality rates from IHD and CBVD with the SDI in 2010 are shown in Figures 2c and 3c, respectively, while the comparisons with the SDI in 2019 are shown in figures 2d and 3d, respectively.

**Figure 2:** Comparisons of percentage variations in standardized mortality rates from ischemic heart disease from 2000 to 2019 with social vulnerability index in 2010 (a), social vulnerability index in 2017 (b), sociodemographic index in 2010 (c), and sociodemographic index in 2019 (d).

**Figure 3:** Comparisons of percentage variations in standardized mortality rates from cerebrovascular disease from 2000 to 2019 with social vulnerability index in 2010 (a), social vulnerability index in 2017 (b), sociodemographic index in 2010 (c), and sociodemographic index in 2019 (d).

Figure 4 shows percentage variations in mortality rates for IHD and CBVD standardized by age group compared with percentage variations in SVI and SDI from 2000 to 2019.

**Figure 4:** Comparisons of percentage variations in standardized mortality rates from ischemic heart disease in the period from 2000 to 2019 with percentage variations in the social vulnerability index (a) and the sociodemographic index (b) in the same period; comparisons of percentage variations in standardized mortality rates from cerebrovascular disease in the period from 2000 to 2019 with percentage variations in social vulnerability index (c) and sociodemographic index (d) in the same period.

## Discussion

The present study showed that, during the analyzed period, IHD became the leading cause of mortality in the country, as demonstrated in previous studies.^6,8,24^ Additionally, although the crude mortality rates from IHD and CBVD increased, due to the demographic transition that occurs in the country, the standardized mortality rate by age group decreased for both conditions. There were also improvements in SDI and SVI, with a predominance of the best indicators in FUs in the South, Southeast, and Midwest regions. When comparing the percentage variations in standardized mortality rates from IHD and CBVD with the SVIs in 2010 and 2017 and the SDIs in 2010 and 2019, the FUs with the best indicators had a greater percentage reduction in mortality. Finally, when comparing the percentage variations in SDI in the period with the percentage variations in standardized mortality rates from IHD and CBVD, the FUs in the South, Southeast, and Midwest regions had the greatest percentage reduction in mortality rates, despite a smaller percentage variation in SDI, which may have occurred because the FUs in these regions started from relatively high SDI values. However, a pattern was not identifiable when this comparison was performed using the percentage variation in SVI. Also of note is that the FUs of Rio de Janeiro, São Paulo, Distrito Federal, and Santa Catarina showed deterioration in the SVI.

Of note, some FUs in the North and Northeast regions had an important positive variation in SDI and a negative variation in SVI, which did not translate into a reduction in mortality from IHD and CBVD, suggesting that there may be a minimum value needed to be reached for the effects to be observed. This effect has already been identified with another index of average human development (MHDI).^7^

This study differs from others published to date on this topic,^7,8,10,15,19,20^ as it proposes to analyze the comparison in mortality rates from IHD and CBVD associated with two socioeconomic indicators that complement each other, *i.e.*, the SDI, which evaluates the degree of social development in a country or region, and the SVI, which fulfills the role of identifying the most vulnerable regions. Additionally, an analysis of the percentage variation of rates and indicators was carried out in order to compare each FU to itself, something that has not been done to date.

The pathophysiological understanding of the influences of classic risk factors in cardiovascular diseases, such as hypertension, dyslipidemia, diabetes mellitus, obesity, smoking, and sedentary lifestyle, have been – and are still – fundamental^17,18^ in guiding measures for prevention and mortality reduction. However, previous studies at a global level have identified differences in trends in mortality rates from IHD and CBVD between countries with different socioeconomic levels.^10,23,24^ Some studies have even shown that countries with higher socioeconomic levels have a higher incidence of noncommunicable diseases – a group that includes CVDs – due to increased exposure to classic risk factors and greater availability of diagnostic and therapeutic methods. Despite this, the probability of deaths from these conditions is higher in countries with lower socioeconomic status.^11,12,25,26^

In the context of CBVD, previous studies have identified the trend toward reduced mortality in the country.^7,8,13,27,28^ This reduction in mortality has been previously identified as occurring heterogeneously, with the North and Northeast regions showing less evident declines and some FUs even showing increasing mortality.^7,8,29^ A similar pattern has also been observed previously regarding IHD.^9,21,24^

The factors that contributed to improved mortality rates from these conditions nationwide include greater access to health services and adoption of prevention strategies,^7,8,30,31^ with the expansion of primary health care^7,32,33^ and development of a strategic action plan for noncommunicable chronic diseases.^7,34^ With CBVD in particular, there was also the creation of a network for initial care of stroke.^7,35,36^ However, these improvements occurred in a heterogeneous way across the country. Added to this is the fact that large centers, which are located mainly in the South and Southeast regions, have access to greater availability of medications and interventional treatments,^6^ along with better levels of schooling and human development.^8,9,14,37^

Among plausible justifications for worsening mortality rates from IHD and CBVD in the FUs in the North and Northeast regions, despite improvement in socioeconomic indicators, are the underreporting of mortality from these conditions, which was higher in these regions, especially at the beginning of the analyzed period,^7,38^ and the demographic transition occurring later in the North and Northeast regions of the country.^7,39^

The main limitations of the study include its observational design, which makes it a “hypothesis generator.” Also, the study was based on a database and is thus subject to bias due to data collection failures, *i.e.*, underreporting, ill-defined causes, or “ garbage codes.” However, these limitations were systemic and occurred across all death certificates and databases, so the limitations are not an impediment to the global data analysis.

Therefore, the results of this study add to those of previous studies^7-9,19,20^ on this topic by suggesting that the improvement in socioeconomic conditions, such as income, work, education, and access to urban infrastructure, have an impact in reducing mortality from CVDs.

## Conclusion

The concomitant analysis of SDI and SVI allowed a more comprehensive evaluation of the socioeconomic profile in Brazil and its FUs and the evaluation of a possible relationship between these indicators and mortality from IHD and CBVD. There was an improvement in human development and a reduction in social vulnerability in the country, associated with a decrease in standardized mortality rates from IHD and CBVD by age group. However, this occurred heterogeneously across the country's territory. The best socioeconomic indicators and the lowest mortality rates from IHD and CBVD were concentrated in the country's South, Southeast, and Midwest regions. This result suggests that greater social development and lower social vulnerability may be related to lower mortality from IHD and CBVD, although the present study could not identify a direct relationship between these indicators and mortality rates.
